# Differences in Clinical Presentation of COVID-19 in Children Hospitalized During Domination of Early (BA.1, BA.2) and Late (BA.5, BA.2.75, BQ.1 and XBB.1.5) SARS-CoV-2 Omicron Subvariants

**DOI:** 10.1097/INF.0000000000004167

**Published:** 2023-11-03

**Authors:** Maria Pokorska-Śpiewak, Małgorzata Pawłowska, Przemysław Ciechanowski, Michał Peregrym, Anna Dobrzeniecka, Małgorzata Sobolewska-Pilarczyk, Justyna Franczak, Ewa Majda-Stanisławska, Barbara Szczepańska, Izabela Zaleska, Robert Flisiak

**Affiliations:** * From the Department of Children’s Infectious Diseases, Medical University of Warsaw, Warsaw, Poland; † Department of Pediatric Infectious Diseases, Regional Hospital of Infectious Diseases in Warsaw, Warsaw, Poland; ‡ Department of Infectious Diseases and Hepatology, Faculty of Medicine, Collegium Medicum, Nicolaus Copernicus University, Bydgoszcz, Poland; § Department of Pediatrics and Infectious Diseases, Regional Hospital in Szczecin, Szczecin, Poland; ¶ Department of Pediatric Infectious Diseases, Medical University of Lodz, Lodz, Poland; ∥ Department of Pediatrics, Pediatric Surgery and Otorhinolaryngology, Collegium Medicum Jan Kochanowski University, Kielce, Poland; ** Department of Pediatrics and Infectious Diseases, Wroclaw Medical University, Wroclaw, Poland; †† Department of Infectious Diseases and Hepatology, Medical University of Bialystok, Bialystok, Poland.

**Keywords:** children, COVID-19, Omicron, SARS-CoV-2, severity

## Abstract

This study analyzed data for 1098 children: 575 diagnosed with COVID-19 between January and June 2022 (early Omicron) and 523 hospitalized from July 2022 to April 2023 (late Omicron). New Omicron subvariants lead to similar recovery rates without deaths and acute respiratory distress syndrome in children as BA.1 and BA.2, however, they more often cause fever and croup. Children suffering from comorbidities, presenting with pulmonary lesions and older, may be prone to a more severe consequences of COVID-19 in terms of the currently dominating Omicron subvariants.

Despite the fact that the clinical course of coronavirus disease 2019 (COVID-19) is milder in children, an increasing proportion of pediatric patients have been observed among reported cases and hospital admissions due to COVID-19 since January 2021.^1^ One of the possible explanations for this observation was the emergence of more transmittable severe acute respiratory syndrome coronavirus 2 (SARS-CoV-2) variants of concern, including Alpha through Delta and Omicron.^2^ The most recent lineage is Omicron (B.1.1.529), which has been dominating in Europe since early 2022.^3^ At the beginning, the BA.1 and BA.2 subvariants prevailed, but they have been replaced by BA.5, BA.2.75 and recombinant sublines BQ.1 and XBB.1.5 in the second half of 2022 (see Figure, Supplemental Digital Content 1, http://links.lww.com/INF/F297).^2,3^ In adult patients, infection with the Omicron variant was associated with a less severe course of COVID-19, with lower rates of hospitalization and intensive care unit (ICU) admissions, as well as decreased mortality rates compared to the previously dominating SARS-CoV-2 variants.^4,5^ There are only scarce data in children analyzing the clinical course of COVID-19 during the Omicron domination and they mainly describe the first months of the Omicron occurrence.^6–12^ Predictors of severe COVID-19 caused by currently dominating variants need to be established in pediatric patients. Thus, the aim of this study was to analyze the clinical course and severity of COVID-19 in children during the dominance of early and later Omicron subvariants to identify predictors of severe course of the disease in the pediatric population.

## MATERIAL AND METHODS

### Study Design

This retrospective study analyzed data collected in a multicenter, nationwide, observational SARSTer database supported by the Polish Association of Epidemiologists and Infectiologists. SARSTer database includes epidemiological and clinical data on both adult and pediatric patients who have been hospitalized due to COVID-19 in 30 Polish inpatient centers that reported their consecutive cases using an electronic questionnaire since the onset of the pandemic. Diagnosis of COVID-19 was based on a second-generation antigen testing or a positive real-time polymerase chain reaction on a nasopharyngeal swab performed in certified diagnostics laboratories. All children were managed according the Polish recommendations for children with COVID-19, which did not alter during the studied period. In this analysis, we included all patients 0–18 years of age, who were diagnosed with COVID-19 between January 1, 2022 and April 30, 2023. For this study, we divided the analyzed period into 2 parts: January 2022–June 2022 (corresponding to early Omicron, EO, when subvariants BA.1 and BA.2 dominated) and July 2022–April 2023 (corresponding late Omicron, LO, with a dominance of subvariants BA.5, BA.2.75, BQ.1 and XBB.1.5). The subvariants dominating in Poland were established according to the Global Initiative on Sharing All Influenza Data (see Figure, Supplemental Digital Content 1, http://links.lww.com/INF/F297).^3^ Children referred to the hospital and released home at the same day after medical assessment, not requiring further hospital stay, were not excluded from this study.

Epidemiologic data included age, sex, comorbidities and vaccination against COVID-19 status. All symptoms at the time of admission and during hospitalization were recorded. The clinical course of the disease was assessed on admission to the hospital and after 7, 14, 21 and 28 days using a scale based on the World Health Organization recommendations, modified to an 8-point version, which has been previously used in adult patients in the SARSTer database.^13,14^

### Statistical Analysis

All statistical analyses were performed using MedCalc Statistical Software version 22.007 (MedCalc, Ostend, Belgium, https://www.medcalc.org). A 2-sided *P* < 0.05 was considered significant. Continuous variables were tested for normal distribution using the Kolmogorov–Smirnov test, and they were expressed as the medians with interquartile ranges or mean ± standard deviations, as appropriate. These data were compared using the Mann–Whitney test or Student’s *t* test. Categorical variables were presented as numbers with percentages and were compared using the *χ*^2^ test. To identify predictors of severe course of COVID-19 in the study group, regression analyses were conducted. A multiple regression was performed with the following variables entered into the model irrespective of the results of the univariate analysis: sex, age, presence of any comorbidity, presence of pulmonary lesions on radiological examination. Separate models were constructed for different indicators of disease severity, for example, need for oxygen therapy or duration of hospitalization >7 days. The results were presented as odds ratios and 95% confidence intervals. Results with confidence intervals not including 1.0 were considered statistically significant.

## RESULTS

### Patients

We identified 1098 patients eligible for this study: 575 during the EO, and 523 in the LO. There was a slight predominance of boys in both analyzed periods. Infants constituted for over 40% of the study participants, and around 80% of children were below 6 years of age, without any significant difference between the studied periods (Table 1). In total, 19.7% of patients suffered from comorbidities, more frequently during the LO compared with EO (23.3% vs. 16.8%, *P* = 0.01). The most reported (19.6%) were viral coinfections (mainly with influenza or respiratory syncytial virus), followed by allergies, endocrine and cardiologic disorders (see Figure, Supplemental Digital Content 2, http://links.lww.com/INF/F298). The vaccination status against COVID-19 was unknown for over a half of the participants. Proportion of children immunized with at least primary vaccination did not exceed 1.5% (Table 1).

**TABLE 1.** Baseline Characteristics of Children Hospitalized Due to COVID-19 During Domination of Early and Late SARS-CoV-2 Omicron Subvariants

Characteristics	Total N = 1098	Early Omicron N = 575	Late Omicron N = 523	*P* Early vs. Late
Sex (girls/boys)	504/594 (45.9/54.1)	257/318 (44.7/55.3)	247/276 (47.2/52.8)	0.40
Age (years) median (IQR)	1.0 (1.0; 3.0)	1.0 (1.0; 3.0)	1.0 (1.0; 3.0)	0.03
<1 year	466 (42.5)	238 (41.4)	228 (43.6)	0.27
1–5 years	434 (39.5)	220 (38.3)	214 (40.9)	
6–10 years	90 (8.2)	51 (8.9)	39 (7.5)	
11–15 years	79 (7.2)	47 (8.2)	32 (6.1)	
16–18 years	29 (2.6)	19 (3.3)	10 (1.9)	
Comorbidities (all)	219 (19.9)	97 (16.8)	122 (23.3)	0.01
Chronic comorbidities	176 (16.0)	76 (13.2)	100 (19.1)	0.007
Coinfections	43 (3.9)	21 (3.6)	22 (4.2)	0.6
Vaccination against COVID-19				
Vaccinated	13 (1.1)	9 (1.5)	4 (0.7)	0.14
Not vaccinated	457 (41.6)	226 (39.4)	231 (44.2)	
Unknown	628 (57.3)	340 (59.1)	288 (55.1)	
Clinical status at the admission to the hospital				
SpO_2_ (%), mean ± SD	97.4 ± 2.3	97.9 ± 1.6	96.8 ± 2.9	<0.0001
Asymptomatic	11 (1.0)	8 (1.4)	3 (0.6)	0.32
Stable symptomatic with SpO_2_ > 95%	1005 (91.5)	526 (91.5)	479 (91.6)	
Unstable symptomatic with SpO_2_ 90%–95%	31 (2.8)	12 (2.1)	19 (3.6)	
Unstable symptomatic with SpO_2_ ≤ 90%	15 (1.3)	8 (1.4)	7 (1.3)	
ARDS	0	0	0	
Unknown	36 (3.2)	21 (3.7)	15 (2.9)	

Data are presented as n (%), unless otherwise indicated.

ARDS indicates acute respiratory distress syndrome; SpO_2_, peripheral oxygen saturation.

### Clinical Presentation at Admission

**FIGURE 1.**
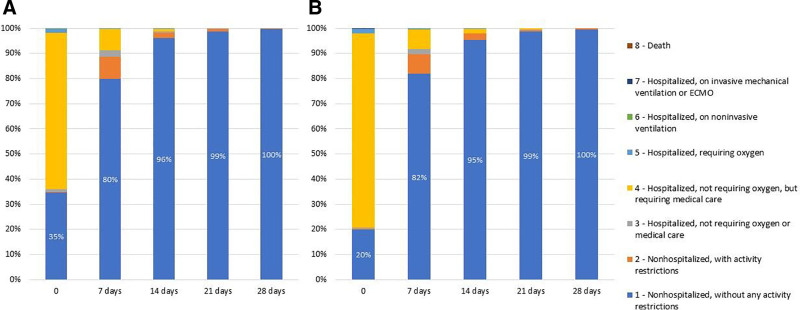
Clinical status of children hospitalized during the domination of early (A) and late (B) SARS-CoV-2 Omicron subvariants at admission to the hospital (0), and after 7, 14, 21 and 28 days. Data are presented as proportions of children presenting with specific clinical status.

**FIGURE 2.**
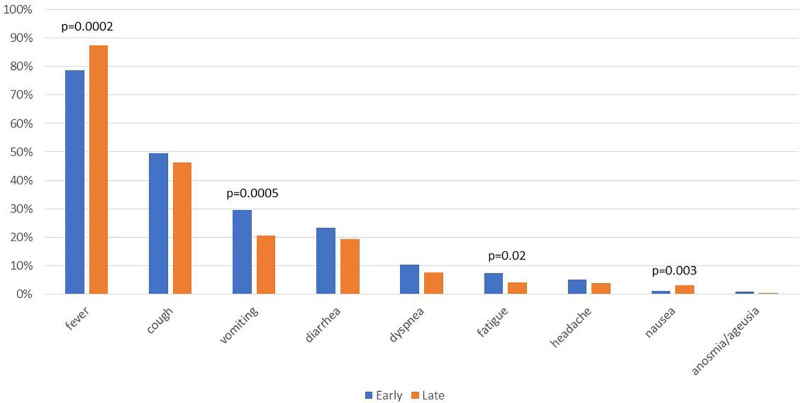
Clinical presentation of COVID-19 in children hospitalized during the domination of early and late SARS-CoV-2 Omicron subvariants. Data are presented as prevalence of clinical symptoms (%). For significant differences, *P* values are provided.

Most children presented with peripheral oxygen saturation (SpO_2_) >95% (Table 1). Most admitted participants were in stable, but symptomatic condition. They usually did not require oxygen therapy, but needed medical symptomatic care (Table 1, Fig. 1). Less than 5% of patients were unstable, with decreased SpO_2_ levels (Table 1). Among the most frequently reported symptoms of COVID-19 were fever and cough (Fig. 2). The prevalence of dyspnea was low and did not exceed 10%. Fever was more commonly reported during the LO compared to EO (87% vs. 79%, *P* = 0.0002), whereas vomiting, fatigue and nausea were less frequent during LO (Fig. 2). Febrile seizures as a complication of COVID-19 occurred among 0.87% of children hospitalized during EO and 1.5% during LO period (*P* = 0.31). Croup was diagnosed more frequently during LO compared to EO period (3.0% vs. 1.2%; *P* = 0.03).

### Laboratory Testing and Radiologic Examinations

Laboratory abnormalities found in the study group were presented in Table, Supplemental Digital Content 3, http://links.lww.com/INF/F299. There were no differences in laboratory testing during both periods. Interestingly, despite a mild clinical course of the disease, a significant proportion of children presented with increased inflammatory markers (leukocytosis, elevated C-reactive protein, procalcitonin, Interleukin-6 and D-dimer, Table, Supplemental Digital Content 3, http://links.lww.com/INF/F299).

Radiologic examination (most commonly chest radiograph, CXR, followed by lungs ultrasonography and computed tomography) was based on the clinical indications and was performed in 105/575 (18.2%) of patients during EO and 103/523 (19.7%) during LO (see Table, Supplemental Digital Content 4, http://links.lww.com/INF/F300). It revealed radiologic lesions in 23/105 (21.9%) and 34/103 (33.0%) of patients, respectively (*P* = 0.07). Thus, pulmonary lesions were found in 23/575 (4.0%) of all children admitted during EO, compared with 34/523 (6.5%) during LO (*P* = 0.06). For clarity, children with coinfections were excluded from this part of analysis.

### Disease Severity and Treatment

Mean duration of hospitalization was longer during LO compared to EO period, but the proportion of children requiring prolonged hospitalization (>7 days) did not differ significantly (Table 2). Around 1% of all participants required rehospitalization. No cases of death, acute respiratory distress syndrome (ARDS) or ICU admission were reported. Oxygen therapy was implemented in 1.4% and 1.7% of patients during EO and LO, respectively. One 17-year-old patient hospitalized in LO period required mechanical ventilation. Children were treated mainly symptomatically, and only a small proportion was treated with antiviral agents: remdesivir was implemented more frequently during LO compared with EO (1.9% vs. 0.5%, *P* = 0.03), whereas only 1 patient received molnupiravir during LO.

**TABLE 2.** Treatment of COVID-19 and Indicators of Disease Severity in Children Hospitalized During Domination of Early and Late SARS-CoV-2 Omicron Subvariants

Treatment/Feature	Early Omicron N = 575	Late Omicron N = 523	*P* Early vs. Late
Duration of hospitalization (days), median (IQR)	3.0 (0; 4.0)	3.0 (1.0; 4.0)	0.01
Hospitalization duration of >7 days	39 (6.7)	29 (5.5)	0.39
Rehospitalization	5 (0.9)	6 (1.2)	0.64
Death	0	0	–
Oxygen therapy	8 (1.4)	9 (1.7)	0.65
Mechanical ventilation	0	1 (0.2)	0.29
Remdesivir	3 (0.5)	10 (1.9)	0.03
Molnupiravir	0	1 (0.2)	0.29
Azithromycin	14 (2.4)	22 (4.2)	0.09
Heparin	2 (0.3)	1 (0.2)	0.62
Dexamethasone	21 (3.7)	9 (1.7)	0.05

Data are presented as n (%), unless otherwise indicated.

### Clinical Course and Its Predictors

The full recovery of all patients was observed within 28 days after admission, including around 80% after 7 days, 95% after 14 days and 99% after 21 days, similarly during both studied periods (Fig. 1). Regression analysis revealed that during EO period, presence of pulmonary lesions was associated with a 7-fold increase in the need for oxygen therapy, whereas the presence of comorbidities increased the risk for prolonged hospitalization (see Table, Supplemental Digital Content 5, http://links.lww.com/INF/F301). During LO, presence of comorbidities and radiological pulmonary lesions were independent predictors of the need for oxygen therapy and prolonged hospitalization (see Table, Supplemental Digital Content 5, http://links.lww.com/INF/F301). In addition, older age was associated with the increased need for oxygen therapy.

## DISCUSSION

In this study, we demonstrated differences in the clinical course and severity of COVID-19 in children hospitalized during domination of early and late SARS-CoV-2 Omicron subvariants. We documented a generally mild clinical presentation and 100%-full recovery rates irrespective of the studied period. There were no cases of death, ARDS and ICU admission, whereas rates of mechanical ventilation or admission >7 days were very low. However, the new Omicron subvariants were more likely to cause fever and croup compared to the initial BA.1 and BA. 2 subvariants. In addition, the mean duration of hospitalization and the need for antiviral treatment increased during their dominance.

Compared to the Delta variant, the occurrence of Omicron in early 2022 was associated with a milder clinical course of COVID-19 and lower mortality rates in adults, despite its higher infectivity and resistance to acquired immunity.^13,15,16^ Newer variants, due to their higher transmissibility have disproportionally affected unvaccinated people and other vulnerable populations including children.^11^ In our cohort, the number of vaccinated participants was very low and did not exceed 1.5%. This is concordant with the reports of the European Centre for Disease Prevention and Control on the COVID-19 vaccination, according to which, until May 2023 in Poland, the primary vaccination uptake was only 0.2% in children 0–4 years, and 17.6% in children 5 to 9 years of age.^17^ On the other hand, a study performed in Poland between June 2021 and April 2022 on 686 children revealed, that the seroprevalence of SARS-CoV-2 IgG antibodies in children hospitalized for reasons other than COVID-19 was 57%, and in April 2022 it was even 87.5%.^18^

In the United States, rates of hospitalization in children 0–4 years old during the initial Omicron wave were even 5 times higher than during Delta variant dominance, but with milder severity of the disease.^19,20^ However, another study reported similar rates of pediatric ICU admissions during Omicron and previous waves of COVID-19.^11^ Observations from Japan showed that with the emergence of Omicron, the number of children hospitalized due to COVID-19 increased significantly compared to the period of Alpha and Delta predominance.^21^ In addition, infection with Omicron BA.1 and BA.2 subvariants was associated with more frequent upper respiratory symptoms and neurological complications.^6,8,21^ Similar observations were made by authors from South Korea, who documented a higher proportion of patients with fever (89.9%), febrile seizures (8.8%) and croup (13.4%).^9^ In our cohort, fever was significantly more frequent during LO period (87% compared to 79% during EO), but the prevalence of febrile seizures was low and did not differ according to the analyzed periods (1.5% during LO, and 0.87% during EO). Fever was found by other authors to occur more frequent during the Omicron wave compared to previous variants,^8,9,11^ which may lead to an increase in the incidence of febrile seizures. In addition, most studies reported that children suffering from COVID-19 during the Omicron wave were significantly younger than in previous periods,^10–12^ which may contribute to a more frequent occurrence of febrile seizures or croup, which are typically seen more often in younger children. The median age among our cohort was 1.0 years, with younger participants during LO than EO, which is significantly lower compared to our previously reported patients hospitalized in 2020 and 2021 (6.8 and 4.1 years, respectively).^22^ Croup prevalence was found to increase during LO, with decreasing age of the participants. More common upper respiratory infection because of Omicron infection was also reported by other authors.^9,10^ By contrast, with an increase in upper respiratory tract involvement during the Omicron wave, a decrease in COVID-related pneumonia was reported.^9^ In our study, radiological pulmonary lesions typical for COVID-19 were found in 4.0% of children admitted during EO, and 6.5% during LO. This suggests a significant decrease in COVID-related pneumonia in comparison to our patients reported during previous waves, 23% during the first months of the pandemic, 15% of patients hospitalized in 2020 and 22% in 2021.^22,23^ In a study by Seriakova et al,^12^ the frequency of CXR changes decreased with subsequent waves of the pandemic and was lowest during the early Omicron dominance (55%), which is however, higher compared to our results. Despite a relative low frequency of pulmonary lesions in our cohort, a significant number of patients presented with elevated inflammatory markers. Data obtained by other authors analyzing the laboratory findings in children hospitalized during the early Omicron period are inconsistent.^10,12^

Based on our observations, predictors of severe course of COVID-19 in children in terms of the currently dominating Omicron subvariants include comorbidities, radiological pulmonary lesions and older age. This is consistent with the observations of Quintero et al.,^11^ who analyzed the severity of COVID-19 by infecting variant and concluded that presence of underlying conditions and viral coinfections, but not the infecting variant of SARS-CoV-2, was associated with worse clinical outcomes. Thus, it seems that despite distinct clinical manifestations during the dominance of subsequent variants, clinical risk factors remain important determinants of COVID-19 severity in children irrespective of the wave of pandemic.^11,24^

One Japanese study compared the clinical characteristics of children infected with SARS-CoV-2 Omicron variant BA.5 (July–August 2022) and BA.1/BA.2 (January–June 2022) in 13 hospitals.^6^ The authors concluded that the emergence of BA.5 had a more severe impact on children, as the incidence of hospitalizations and neurological complications (including febrile seizures with prevalence of 27.9% vs. 7.0% during BA.1/BA.2) increased significantly.^6^ Thus, despite the overall good prognosis for children suffering from COVID-19 during LO, it should be considered that the impact of the new Omicron subvariants on pediatric patients seems to be different than in adults, in which better clinical outcomes were observed according to the data from SARSTer database.^25^ In addition, selected cases, in particular older children with comorbidities and with confirmed radiological pulmonary lesions in the course of SARS-CoV-2 infection, should be managed with precaution.

Our study is limited by its retrospective design, which might lead to lack of some data and observational bias. Secondly, the study had been conducted in hospital settings, thus, the spectrum of pediatric COVID-19 may have been affected by the patient population, representing more severely ill children. In addition, we did not verify the virus strain by molecular diagnostics methods, as such testing is not available for clinical purposes. However, we were basing our results on the available objective and most reliable epidemiological datasets. In addition, due to a limited number of patients presenting with different comorbidities, we did not analyze the influence of particular underlying conditions on the disease severity. Moreover, laboratory abnormalities were only presented as proportions of tests exceeding upper or lower limit of normal, and were not further analyzed. However, to the best of our knowledge, we present one of the most prominent pediatric national European cohorts documenting the differences in COVID-19 clinical course during EO and LO dominances.

In conclusion, the new Omicron subvariants BA.5, BA.2.75, BQ.1 and XBB.1.5, which have been prevailing since mid-2022, lead to similar recovery rates in children as BA.1 and BA.2 subvariants, with similar prolonged hospitalization and rehospitalization rates, and equally rare ARDS and deaths. However, they more often cause fever and croup, which may be also associated with the younger age of hospitalized children. In addition, the mean duration of hospitalization and the need for antiviral treatment increased during their dominance. In particular, children suffering from comorbidities (both chronic and coinfections), presenting with radiological pulmonary lesions and older, may be prone to a more severe consequences of COVID-19 in terms of the currently dominating Omicron subvariants.

### Ethical Statement

Ethical review and approval were waived because the study was retrospective, noninterventional and based on data collected in the national SARSTer database. Therefore, it does not require approval of the ethics committee. Due to the retrospective nature of the presented study, written consent by participants was waived. The patients’ data was protected according to the European Union General Data Protection Regulation.

## Supplementary Material


