# Supplementary material for: Differences in Clinical Presentation of COVID-19 in Children Hospitalized During Domination of Early (BA.1, BA.2) and Late (BA.5, BA.2.75, BQ.1 and XBB.1.5) SARS-CoV-2 Omicron Subvariants
Source: Pediatr Infect Dis J. 2023 Nov 3;43(2):149–54. doi: 10.1097/INF.0000000000004167 (PMC11500694; doi:10.1097/INF.0000000000004167)
Supplement: Supplementary file 1 [file inf-43-149-s001.docx]

**Supplemental Digital Content 1.** Genomic epidemiology of SARS-CoV-2 with subsampling in Europe since the onset of the pandemic. We indicated the analyzed periods of domination of early and late SARS-CoV-2 Omicron subvariants (according to Gisaid.org, <https://nextstrain.org/ncov/gisaid/europe/all-time?dmax=2023-04-30&f_region=Europe>, accessed at 22.07.2023)

**
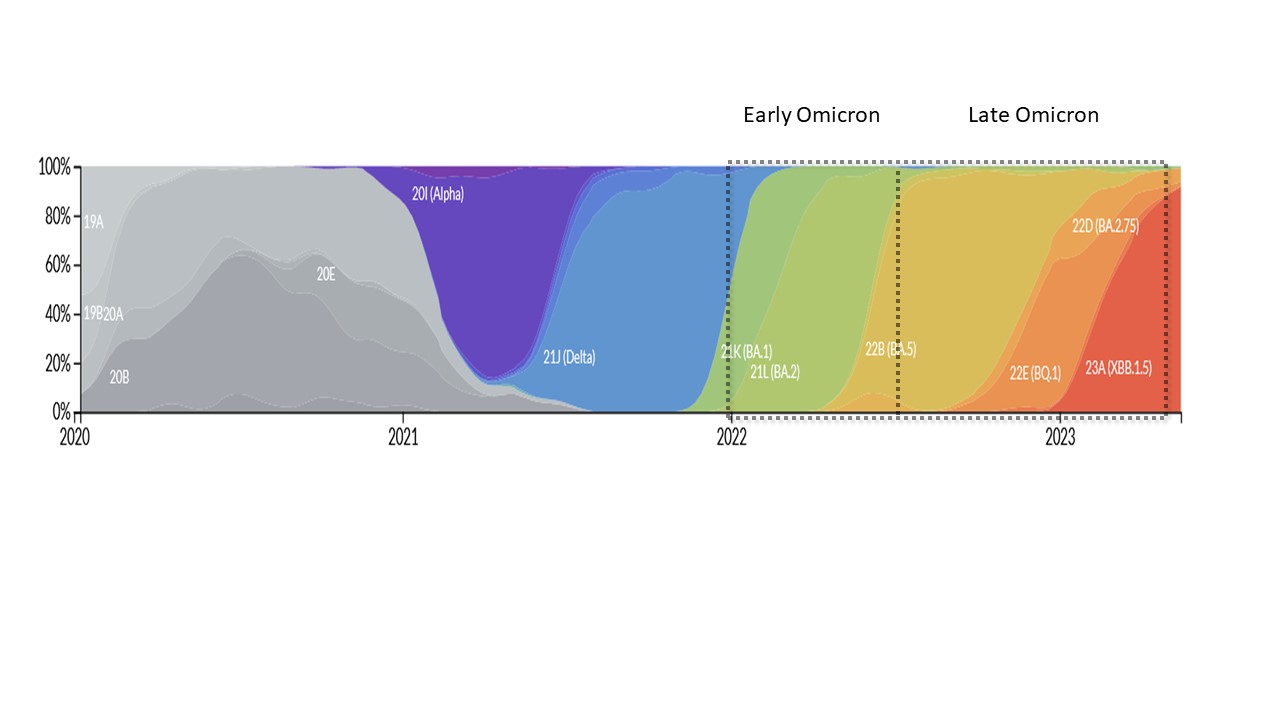
**
