# Supplementary material for: Differences in Clinical Presentation of COVID-19 in Children Hospitalized During Domination of Early (BA.1, BA.2) and Late (BA.5, BA.2.75, BQ.1 and XBB.1.5) SARS-CoV-2 Omicron Subvariants
Source: Pediatr Infect Dis J. 2023 Nov 3;43(2):149–54. doi: 10.1097/INF.0000000000004167 (PMC11500694; doi:10.1097/INF.0000000000004167)
Supplement: Supplementary file 2 [file inf-43-149-s002.docx]

**Supplemental Digital Content 2.** Comorbidities among children hospitalized due to COVID-19 during domination of early and late SARS-CoV-2 Omicron subvariants. Data are presented as proportion (%) of a total of 219 participants presenting with any comorbidity.

*Coinfections included: 9 cases of respiratory syncytial virus (RSV) infection; 8 cases of influenza; 16 cases of bacterial infections; and 10 cases of other viral infections.
