# Supplementary material for: Differences in Clinical Presentation of COVID-19 in Children Hospitalized During Domination of Early (BA.1, BA.2) and Late (BA.5, BA.2.75, BQ.1 and XBB.1.5) SARS-CoV-2 Omicron Subvariants
Source: Pediatr Infect Dis J. 2023 Nov 3;43(2):149–54. doi: 10.1097/INF.0000000000004167 (PMC11500694; doi:10.1097/INF.0000000000004167)
Supplement: Supplementary file 3 [file inf-43-149-s003.docx]

**Supplemental Digital Content 3.** Laboratory findings in children hospitalized due to COVID-19 during domination of early and late SARS-CoV-2 Omicron subvariants

| **Laboratory abnormality** | **Early Omicron** | **Late Omicron** | **P**  **Early vs. Late** |
| --- | --- | --- | --- |
| Leukocytosis (> 12 000/mm^3^) | 90/418 (21.5) | 83/375 (22.1) | 0.83 |
| Leukopenia (< 4 000/mm^3^) | 26/418 (6.2) | 34/375 (9.1) | 0.13 |
| Thrombocytosis (> 450 G/L) | 32/415 (7.7) | 35/372 (9.4) | 0.39 |
| Thrombocytopenia (< 120 G/L) | 18/415 (4.3) | 15/372 (4.0) | 0.83 |
| Elevated C-reactive protein (> 10 mg/L) | 145/415 (34.9) | 143/376 (38.0) | 0.36 |
| Elevated procalcitonin (> 0.5 ng/mL) | 52/325 (16.0) | 55/256 (21.4) | 0.09 |
| Elevated Interleukin-6 (> 7 pg/mL) | 172/255 (67.4) | 81/105 (77.1) | 0.06 |
| Elevated D-Dimer (> 500 ng/mL) | 128/243 (52.7) | 86/162 (53.1) | 0.93 |

Data are presented as proportions [n (%)] of children, in which the specific laboratory testing was performed
