# Supplementary material for: Differences in Clinical Presentation of COVID-19 in Children Hospitalized During Domination of Early (BA.1, BA.2) and Late (BA.5, BA.2.75, BQ.1 and XBB.1.5) SARS-CoV-2 Omicron Subvariants
Source: Pediatr Infect Dis J. 2023 Nov 3;43(2):149–54. doi: 10.1097/INF.0000000000004167 (PMC11500694; doi:10.1097/INF.0000000000004167)
Supplement: Supplementary file 4 [file inf-43-149-s004.docx]

**Supplemental Digital Content 4.** Radiologic lesions in children hospitalized during domination of early and late SARS-CoV-2 Omicron subvariants. Children with coinfections were excluded from this analysis.

| **Radiologic examination (any performed)** | **Total**  **N=208** | **Early Omicron**  **N=105** | **Late Omicron**  **N=103** | **P**  **Early vs. Late** |
| --- | --- | --- | --- | --- |
| Lesions detected by any method | 57 (27.4) | 23 (21.9) | 34 (33.0) | 0.07 |
| Lesions detected by CXR | 37 (17.7) | 15 (14.2) | 22 (21.3) | 0.18 |
| Lesions detected by CT | 5 (2.4) | 2 (1.9) | 3 (2.9) | 0.63 |
| Lesions detected by ultrasonography | 18 (8.6) | 8 (7.6) | 10 (9.7) | 0.59 |

Data are presented as number (%). CXR – chest X-ray; CT – computed tomography;
