# Supplementary material for: Differences in Clinical Presentation of COVID-19 in Children Hospitalized During Domination of Early (BA.1, BA.2) and Late (BA.5, BA.2.75, BQ.1 and XBB.1.5) SARS-CoV-2 Omicron Subvariants
Source: Pediatr Infect Dis J. 2023 Nov 3;43(2):149–54. doi: 10.1097/INF.0000000000004167 (PMC11500694; doi:10.1097/INF.0000000000004167)
Supplement: Supplementary file 5 [file inf-43-149-s005.docx]

**Supplemental Digital Content 5.** Indicators of severe course of COVID-19 in children hospitalized during domination of early and late SARS-CoV-2 Omicron subvariants (logistic regression analysis). Data are expressed as odds ratios (OR) 95% Confidence Intervals (CI) and p-value

| **Feature** | **Early Omicron (N = 575)** | | **Late Omicron (N = 523)** | |
| --- | --- | --- | --- | --- |
|  | **OR (95% CI)** | **p** | **OR (95% CI)** | **p** |
| **Need for oxygen therapy** | **N = 8** | | **N = 9** | |
| Age (years) | 1.06 (0.93-1.20) | NS | 1.13 (1.01-1.27) | 0.03 |
| Sex (for male vs. female) | 1.43 (0.31-5.76) | NS | 3.29 (0.67-16.02) | NS |
| Presence of comorbidities | 1.65 (0.32-8.33) | NS | 6.82 (1.68-27.71) | 0.005 |
| Presence of pulmonary lesions in radiological examination | 7.13 (2.09-24.35) | 0.01 | 13.10 (3.78-45.3) | 0.0002 |
| **Hospitalization for > 7 days** | **N = 39** | | **N = 29** | |
| Age (years) | 1.01 (0.94-1.08) | NS | 1.07 (0.99-1.15) | NS |
| Sex (for male vs. female) | 1.17 (0.60-2.27) | NS | 1.65 (0.75-3.66) | NS |
| Presence of comorbidities | 2.31 (1.12-4.74) | 0.02 | 9.55 (4.09-22.32) | <0.0001 |
| Presence of pulmonary lesions in radiologic examination | 1.12 (0.37-3.39) | NS | 6.65 (2.82-15.66) | 0.0001 |
